# Supplementary material for: Value of using artificial intelligence derived clusters by health and social care need in primary care: A qualitative interview study with patients living with multiple long-term conditions, carers and health care professionals
Source: J Multimorb Comorb. 2025 Jun 24;15:26335565251353016. doi: 10.1177/26335565251353016 (PMC12188064; doi:10.1177/26335565251353016)
Supplement: Supplemental Material - Value of using artificial intelligence derived clusters by health and social care need in primary care: A qualitative interview study with patients living with multiple long-term conditions, carers and health care professionals [file sj-pdf-1-cob-10.1177_26335565251353016.pdf]

Appendix 1 – Study adverts

Note:

This document outlines example text and/or images that we will use in our study advertisements. This may take the form of text, images and/or video content (with voiceovers). The adverts may evolve over time to match the developing needs of the study. However, the underlying meaning of the content will remain the same. These materials will include relevant ERGO numbers, dates and version numbers where possible (e.g. on images, in videos). Where wordcount is limited (e.g. on social media), participants will be linked to the study website where all ERGO numbers, dates and version numbers will be visible.

People living with MLTCs

Advertisement for websites/posters:

Do you have two or more health conditions? If yes, we are interested in hearing your views about a new approach to improving health, lifestyle and social care!

We'd like to chat with you on the telephone or online for up to 60 minutes. This will on a convenient date and time for you. We might be able to speak to you in-person, if you'd prefer.

If you are interested or have any questions, please email us at: [study email here] or phone: [study phone number here]

Or go to our website: [study website link]

Advertisement for social media (280 character word limit):

Do you have two or more health conditions?

We are interested in hearing your views about a new approach to improve care. We'd like to chat with you on the telephone or online.

Health and social care professionals

Advertisement for websites/posters:

Are you a health or social care professional who is involved in the healthcare of people with multiple long-term conditions? This includes doctors, nurses but also people who care for someone with multiple long-term conditions (either paid or family carers).

If yes, we are interested in hearing your views about a new approach to improving care by clustering people together who have similar health and social needs.

We'd like to invite you to participate in an interview either on Microsoft Teams or by telephone. We will give you some options of dates and times to make this convenient for you. This interview will last up to 60 minutes.

Advertisement for social media (280 character word limit):

Are you a health or social care professional who is involved in the healthcare of people with long term conditions?

We'd like to hear your views on a new approach to improving care by inviting you to an online or telephone interview.

Appendix 2 – Topic Guide

People living with MLTCs

Lay title: Exploring a new approach to improving health, lifestyle and social care

<u>Introduction</u>

- Explain who you are [*your name, role & that you are part of a team at UoS*].
- We would like to understand your experience living with several different long-term health conditions. We'd also like to know about how your health and lifestyle needs are (or are not) currently being met and why this might be happening. Then we'd like to chat to you about a new approach to support patients getting the help they need for their illnesses, and what your thoughts are on this approach.
- We will use the information from our talk to see if and how care can be improved for people with multiple health conditions in the UK.
- Thank you for completing the online consent form. Did you have any questions?
- As mentioned in our consent form, we will record our conversation so we can listen again to what is being said. Everything we talk about here will be **confidential**. We will take care to make sure that all the information you share with us is kept safely and securely. Your care providers will not know you have spoken with us.
- When I start the audio recorder, I will begin by confirming that you read and completed the online consent form and that you are happy to speak with me today. This is just so

we have a verbal record of your consent, as well as that written version you completed online.

- I will take some notes during our interview, but the recording allows us to know exactly what you've said in your own words. We will remove your name and personal details so people will not be able to identify you.
- You may see me look over to one side during the interview, this is just because I am checking my questions on my other screen.
- The interview will last up to 60 minutes [*check that the interviewee has this time available*].
- The interview can be stopped at any time and without reason [*if this happens, make a note of why they stopped it, if they mention*].
- Before we start, may I please ask whether you are feeling comfortable and have everything you need, such as drinks or snacks, and comfortable room to talk.
- [start audio recorder] Now that I have the audio recorder on, can I confirm that you completed the online consent form for the study and you are happy to take part?

Main Questions	Prompts Questions	Ideas that might be raised by the participant<br><br>These will not be used as prompts/probes but will be for researchers to keep in mind if any of these topics are raised by the participant
**General context**<br><br>Please tell me about what it is like living with different long-term health conditions	• What conditions do you have?<br>• Talk me through anything that stops you from doing what you would like to do day-to-day, related to your health conditions<br>• Tell me about if you've ever sought any support for these issues.<br>• Tell me about any services or support that you think would improve your	Warm up question but move onto next section ASAP – revisiting any ideas that emerge during the general context conversation

	health conditions or your day-to-day life	
**Cluster approach**<br><br>We are looking for ways to help people think about their day-to-day needs when managing their health conditions.<br><br>To do this, routine data in the NHS (such as GP records) would be used to look for patterns to see which people have similar needs or challenges in their	• What do you think of this idea?<br>• How would you feel about being put into a group?<br>• Based on the information you gave on your online survey, our AI would place you in a group as being at risk of developing needs related to [x]. What do you think about that? Does that reflect your experience of having these conditions? What do you think of the word 'risk'?<br>• What would you think if	Prompt around the seven social care needs categories:<br>• ADL<br>• Community<br>• Disability<br>• Finance<br>• Mobility<br>• Residential<br>• Social Network

day-to-day life, to work out what other support they may need and when they might need them. These patterns are going to be created using artificial intelligence (or high tech computers).<br><br>You might have a similar pattern to multiple other people in the data. We call this being in a 'group'. This isn't a group that ever meets, like a social support group. It just means that you have a similar	you were given advice about X social care need? Is that important to you? Why/why not?<br>• How would you feel if you didn't agree with the group you've been put into? (give examples e.g. mental health support if they have self-identified low mental health needs)<br>• What are the problems with this approach? How could we address this?<br>• What are the advantages of this approach? What would encourage you to use this approach?<br>• Would you like to receive information about social care services? Why?	

pattern of data and needs to a group of other people.<br><br>By looking at the patterns in these groups, the NHS can see if people have similarities in their illness over time, and use this to predict what might happen to other people within that group over time.<br><br>The idea of looking at these patterns/ or groups like this, is to provide the right information for different groups more easily, more	• How would you like this information to be given to you? What are your priorities for receiving this information? What services would you like to see? What do you find difficult about that at the moment?<br>• How and what would you like to be told by your GP?<br>• What do you think of a leaflet/website?<br>• Who should be having these conversations with you? Where would be the best place for support?	

quickly and make sure people with needs aren't missed. This grouping system isn't currently used in the NHS.<br><br>These patterns can be used to tailor support for each group. This could take various formats (leaflet, website, conversations with a health care provider)		
Vignette/example (note: there will be several different vignettes to help	• What do you think of that story? How well can you relate to any of it	

to prompt discussion with participants)*	yourself? Do you disagree with any of it? Why?<br>• Have you thought about your own social needs before? Why? And what are they?<br>• Do you feel like you've been able to access the services you need?<br>• Do you think you need more support? What support? Why/what do you find difficult about that at the moment?	
Is there anything else that you would like to say that we haven't discussed so far in this interview?	*Possibly follow up on an interesting point(s) raised by the interviewee, which may have come into your mind or you think is worth revisiting*	

Do you have any questions for us?	• *Do you wish to receive results of the study?*<br>• *Ask the participant if they can **recommend anyone else** who we could interview?*<br>• *Thank participant. Ensure they have **study contact information** for any future enquiries. (Note: prepared some contacts if participants are upset by the interview, such as GP, charity helpline, etc).*<br>• *Voucher? Get address for posting.*<br><br>Close the interview.	

*Vignettes

The purpose of the vignettes is to be able to prompt around the complex idea of cluster-based interventions for improving health and social care in a relatable way for study participants. Vignettes will develop as the clusters are defined (in a separate study within our research team) and as interviews are completed, to address the research questions.

An example vignette is presented here. Where gaps are indicated, demographics similar to the participant will be used. For example, common names used in ethnic groups, similar age range to participant, same gender identity/pronouns as participant etc. This is to ensure that the story presented in the vignette is as relatable as possible.

The vignette has three sections. The first is a general introduction and a usual care scenario. The second is introducing the idea of clusters. The third introduces potential ideas about how clusters might be used in practice to improve health and social care. After each section of the vignette, the research will stop to probe and ask questions.

Example vignette

Section	Vignette	Probes
Section 1<br><br>General introduction and usual care	[name] is a [age] who has [MLTC similar to study participant].<br><br>*They have been experiencing some challenges. [use challenges similar to those mentioned by the participant in the interview]*<br>• *With walking to and from the shops*<br>• *With affording to pay the bills due to being on sickness leave from work*<br><br>*They want help with these challenges but don't know where to start. They decide to ask their GP about this. However, the GP says they will have to discuss these challenges next time, as the appointment has already been taken up by discussing changes needed to medication.*	• What do you think of that story? How well can you relate to any of it yourself? Do you disagree with any of it? Why?<br>• Have you thought about your own social needs before? Why? And what are they?<br>• Have you had conversations like this with your GP before? Why/why not? How did these conversations go?<br>• Do you feel like you've been able to access the services you need?

		• Do you think you need more support? What support? Why?<br>• What did you think of how the GP handled this situation in the story?
Section 2<br><br>Cluster introduction	Here's how the story could have been a bit different.<br><br>At the appointment, the GP asks [name] some questions about their life and how they have been getting on. Based on the answers to these questions, the GP says that [name] fits into a group of people who have similar challenges with their long-term conditions that put them at a risk of their day-to-day life becoming more challenging over time.	• What do you think of this idea?<br>• How would you feel about being put into a group?<br>• Based on the information you gave on your online survey, our AI would place you in a group as being at risk of developing needs related to [x]. What

	By looking at what has helped people similar to [name] in the past, the GP might be able to work out what could be helpful.	do you think about that? Does that reflect your experience of having these conditions? What do you think of the word 'risk'?<br>• What would you think if you were given advice about X social care need? Is that important to you? Why/why not?<br>• What are the problems with this approach? How could we address this?<br>• What are the advantages of this approach? What would encourage you to use this approach?

		• What would you expect the GP to do next?<br>• Would you like to receive information about social care services? Why?<br>• How would you like this information to be given to you? What are your priorities for receiving this information? What information would you like? What do you find difficult about that at the moment?<br>• How and what would you like to be told by your GP?

Section 3<br><br>Ideas about how clusters might be used in practice	The GP provides a leaflet with resources that other people in the same group have found helpful.<br>These resources include social support networks that [name] can get in touch with.	• What do you think of a leaflet? Is this what you expected? Why/why not?<br>• What services would you expect to see in this leaflet? What do you find difficult about that at the moment?<br>• What are the problems with this approach? How could we address this?<br>• What are the advantages of this approach? What would encourage you to use this approach?<br>• What would you expect the GP to do

		next?
